# Genome assembly provides insights into the genome evolution of *Baccaurea ramiflora* Lour.

**DOI:** 10.1038/s41598-024-55498-4

**Published:** 2024-02-28

**Authors:** Jianjian Huang, Jie Chen, Min Shi, Jiaqi Zheng, Ming Chen, Linjun Wu, Hui Zhu, Yuzhong Zheng, Qinghan Wu, Fengnian Wu

**Affiliations:** 1 School of Life Sciences and Food Engineering, Hanshan Normal University, Chaozhou, 521041 Guangdong China; 2 College of Coastal Agricultural Sciences, Guangdong Ocean University, Zhanjiang, 524088 Guangdong China

**Keywords:** *Baccaurea ramiflora*, Whole-genome sequencing, de novo assembly, Genome evolution, Genome, Genomics

## Abstract

*Baccaurea ramiflora* Lour., an evergreen tree of the *Baccaurea* genus of the Phyllanthaceae family, is primarily distributed in South Asia, Southeast Asia, and southern China, including southern Yunnan Province. It is a wild or semi-cultivated tree species with ornamental, edible, and medicinal value, exhibiting significant development potential. In this study, we present the whole-genome sequencing of *B. ramiflora,* employing a combination of PacBio SMRT and Illumina HiSeq 2500 sequencing techniques. The assembled genome size was 975.8 Mb, with a contig N50 of 509.33 kb and the longest contig measuring 7.74 Mb. The genome comprises approximately 73.47% highly repetitive sequences, of which 52.1% are long terminal repeat–retrotransposon sequences. A total of 29,172 protein-coding genes were predicted, of which 25,980 (89.06%) have been annotated, Additionally, 3452 non-coding RNAs were identified. Comparative genomic analysis revealed a close relationship between *B. ramiflora* and the Euphorbiaceae family, with both being sister groups that diverged approximately 59.9 million years ago. During the evolutionary process, *B. ramiflora* exhibited positive selection in 278 candidate genes. Synonymous substitution rate and collinearity analysis demonstrated that *B. ramiflora* underwent a single ancient genome-wide triploidization event, without recent genome-wide duplication events. This high-quality *B. ramiflora* genome provides a valuable resource for basic research and tree improvement programs focusing on the Phyllanthaceae family.

## Introduction

**Figure 1:**
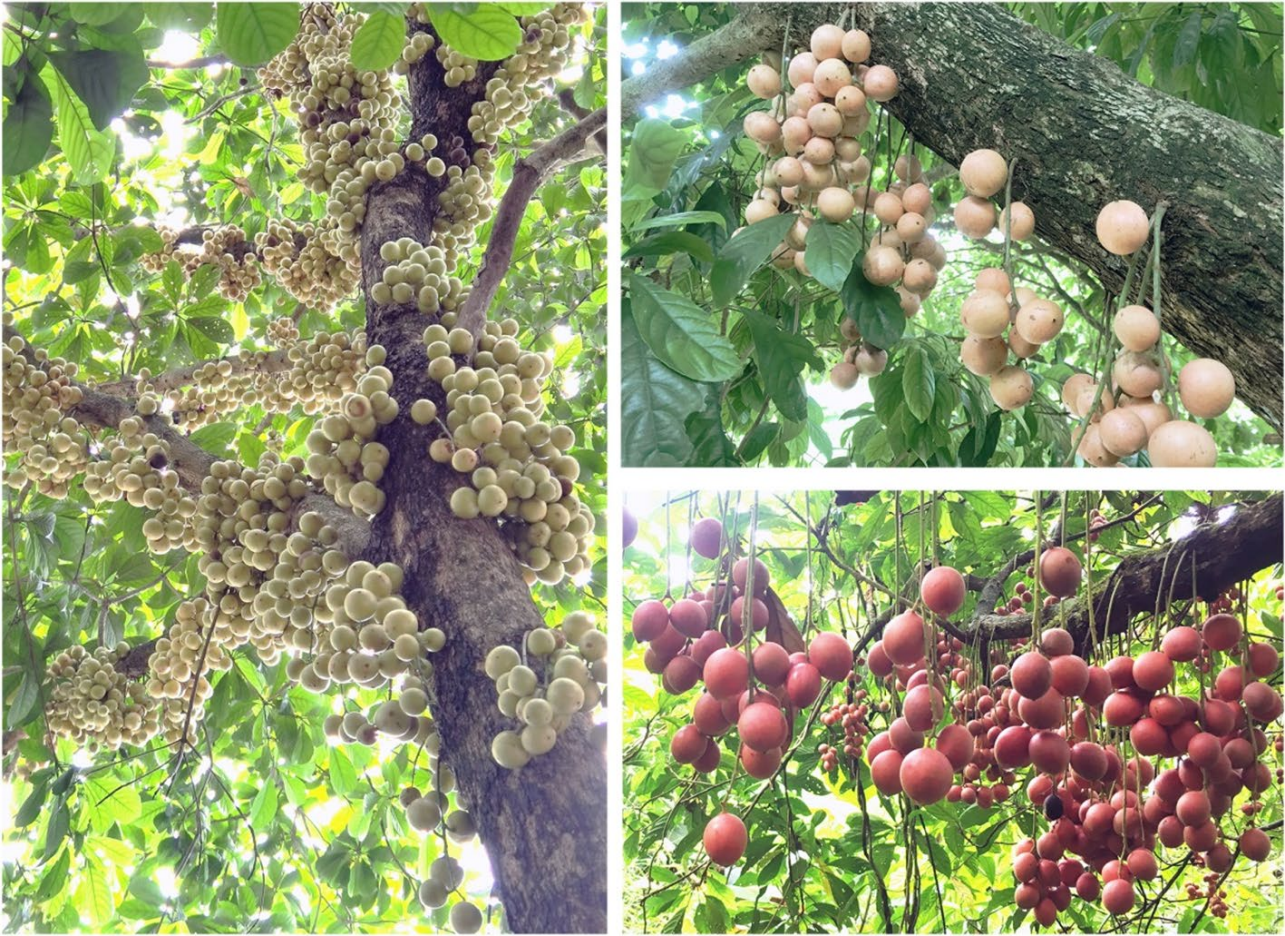
Ripening fruit of *B. ramiflora*.

*Baccaurea ramiflora* Lour., an evergreen tree of the *Baccaurea* genus of the Phyllanthaceae family of the Malpighiales order (APG IV system) (Fig. 1), is distributed in Southeast Asia and South Asia, is mainly cultivated in South China, including in southern Yunnan Province^1,2^, and is occasionally cultivated in Nepal, India, Myanmar, Bangladesh, Indochina, Thailand, and Malaysia^3^. It is an underutilized tropical wild fruit tree species with unique garden and landscape development value. It is a typical tropical plant with old stems that bear flowers, with a beautiful tree shape, several fruit colors (green, white, light yellow, orange, pink, purple, etc.), and different fruit shapes (spherical, long oval, olive, etc.). When fruiting, the fruits grow densely on old branches, making the tree an ideal choice for garden landscaping for its appealing fruits and branches^4^.

The pulp of *B. ramiflora*, sweet and sour, with a tender texture, can be consumed fresh or processed in the forms of fruit juice, preserved fruit, jam, and fruit wine^5^. It is rich in nutrients, with an edible rate of 49.2%, consisting of water (84.7%), carbohydrate (86.14%), fats (0.06%), crude protein (5.43%), fibers (0.29%), vitamin C (1.57 mg/100 g), titratable acid (1.99%), and total sugar (11.87%)^6–9^. Generally, the pulp yield per plant of an adult *B. ramiflora* tree can reach 100–150 kg, so planting *B. ramiflora* can bring many economic benefits to farmers^9^. However, *B. ramiflora* has not yet been commercially planted and utilized^7^, although some landrace trees have been planted around the houses of local farmers. *Baccaurea ramiflora* exhibits significant variations in flowering and fruiting times depending on its origin. For instance, *B. ramiflora* trees from Hainan and Southeast Asia regions bloom and bear fruit approximately 45 days earlier than those in mainland China. The fruits ripen between late May and early August, resembling the ripening period of lychee. Given the diverse flowering and fruiting periods observed in *B. ramiflora* from different provenances, it holds the potential to alleviate the fruit shortage in the current season^5^.

*Baccaurea ramiflora* also has high medicinal and health value^4,10^. Its leaves, fruits, stems, bark and seeds are the ingredients of many Chinese herbal medicines that have long been used to treat jaundice, constipation, indigestion, cellulitis, inflammation, and rheumatoid arthritis, as well as being an antidote for snake venom^2,11–14^. The chemical and medicinal ingredients of *B. ramiflora* have been investigated extensively, and more than 30 compounds that have a host of biological activities such as analgesic, anthelmintic, antioxidant, antidiarrheal, anti-inflammatory, cytotoxic, hemolytic, hypoglycemic, hypolipidemic, insecticidal, neuropharmacological, platelet, antifungal, antibacterial, and other medicinal values have been isolated and characterized^15–20^.

Although *B. ramiflora* is rich in germplasm resources, with diverse fruit phenotypes and varying fruit quality, artificially developed elite varieties have not been reported, and cultivated trees are mostly landraces without a unified evaluation standard. Due to the lack of investigations of the germplasm resources and genomics of *B. ramiflora*, elite strains of *B. ramiflora* have not been developed, and there are no large-scale commercial plantations of the tree^5,7^. Genomic studies of *B. ramiflora* are important for understanding its germplasm resources but have not been conducted yet, which has limited the tree improvement work and thus the tree promotion and production. As a result, *B. ramiflora* breeding is still in its infancy. A complete *B. ramiflora* genome would be of great significance for the study of the diversity of *B. ramiflora* germplasm resources and the mining of functional genes related to agronomic traits. Here, we report the sequencing and assembly of *B. ramiflora* and the analysis of its gene family evolution, whole-genome duplication events, and collinearity. As a reference genome, this *B. ramiflora* genome can provide molecular data for research on the Phyllostachys family.

## Results

### Sequencing and assembly

The sequencing and assembly of the *B. ramiflora* genome were performed based on long-read PacBio SMRT sequencing in combination with short-read Illumina HiSeq2500 sequencing for error correction. Through approximately 60 Gb of next-generation sequencing data, we conducted a survey analysis of the genome with a K-mer analysis of 19 and estimated that its genome size was approximately 973 Mb, with a heterozygosity of 0.634% and a repeat sequence ratio of 57.5% (Fig. S1). The Illumina HiSeq2500 sequencing generated approximately 68.6 Gb of raw reads, with a sequencing depth of 60 × , while the PacBio SMRT sequencing generated 114.14 Gb of raw reads, with a sequencing depth of 100 × . The *B. ramiflora* genome was assembled through different assembly methods (Table S1), including Canu + deredundancy^21^, WTDBG^22^ (Canu error correction + WTDBG assembly), WTDBG + deredundancy^22^, and Flye + deredundancy^23^. The Canu + deredundancy method showed the lowest properly paired mapping rate (69.59%) in the next-gen sequencing data, and WTDBG, Flye, and WTDBG + deredundancy showed obvious abnormal secondary peaks in the GC depth graph (inconsistent with the GC content of the main peak, with an average GC depth of below 20 ×). Considering that the assembly difficulty may be related to the complexity of the species itself, based on the positions of the abnormal peaks in the above assembly results, we proportionally filtered the original subreads, in which subreads with a GC content in the range of 40–50% were filtered out, and the GC content of the filtered subread sequences essentially became normally distributed. The filtered subreads were assembled using the Canu assembler, after which the preliminary assembly did not show any abnormal GC peak, and the genome size after deredundancy was close to the survey evaluation size, with a ContigN50 of approximately 300 kb. To further improve the assembly, after filtering out the subreads highly homologous to mitochondrial and chloroplast sequences, the remaining sequences were assembled using the Canu + deheterozygosity method, which achieved the best assembly, with a normal distribution of GC content and no abnormal peaks.

The size of the final assembly of *B. ramiflora* genome was approximately 975.8 Mb, which was slightly larger than the genome size estimated based on K-mer analysis. This was likely because the high heterozygosity of the genome led two sister chromatids to be assembled together, resulting in a mixed genome greater than the actual size. The assembly contained 3,346 contigs, with the following statistics: GC content: 35.31%; N50: 509.33 kb; Contig Min: 1.63 kb; N90: 118.07 Kb; Contig Max: 7.74 Mb. For plant genomes with high heterozygosity, an assembly with a contig N50 of 509.33 kb is considered good. Compared with the assembled genomes of other plant species of family Euphorbiaceae, i.e., *Ricinus communis* (GenBank accession No.: GCA_000151685.2) (genome size: 350.5 Mb; Contig N50 21.4 kb), *Manihot esculenta* (GCA_001659605.2) (genome size: 693.6 Mb; Contig N50 3.3 Mb), *Hevea brasiliensis* (GCA_001907995.1) (genome size: 1.5 Gb; Contig N50 152.7 kb), and *Jatropha curcas* (GCA_014843425.1) (genome size: 266.8 Mb, Contig N50 134.3 kb) (data source: NCBI database), the genome of *B. ramiflora* is large, being smaller only than that of *H. brasiliensis*, with a Contig N50 only smaller than that of *M. esculenta*.

### Genomic evaluation

To verify the accuracy and completeness of the assembly, we first evaluated the data mapping ratio of the DNA library, which accounted for 98.86% of the total. When we used the RNA libraries of three different tissues of *B. ramiflora* for alignment, the alignment rate was 88.38% for roots, 89.73% for stems, and 90.43% for leaves (Table S2). Finally, we evaluated the *B. ramiflora* genome assembly using the benchmarking universal single-copy orthologs (BUSCO)^24^ and found 2121 BUSCOs, of which 97.5% were single-copy BUSCOs (86.7% were both unique and complete, and 10.8% were multicopy and complete), 0.8% were BUSCOs with incomplete coverage, and 1.7% were missing BUSCOs, indicating that most of the single-copy genes in the genome of *B. ramiflora* are included without repeats or overassembly. The genome assembly is high-quality and has high coverage.

### Gene annotation

The information of assembled repeated sequences of *B. ramiflora* is summarized in Table S3. The assembled repeated sequences accounted for 73.47% (716.93 Mb) of the total sequences, higher than the K-mer analysis estimation (57.5%), indicating the complexity of the *B. ramiflora* genome. Long terminal repeat (LTR)-retrotransposons are a class of transposon with the highest proportion of repeat sequences in plant genomes and the main cause of genome expansion, so high-quality sequences of LTR-retrotransposons are crucial to a genome assembly^25,26^. Sequences of LTR-retrotransposons in the *B. ramiflora* genome were mainly of the types Copia (89.67 Mb, 9.19% of the genome) and Gypsy (281.22 Mb, 28.82% of the genome), and the LTR-retrotransposons of other types were 137.53 Mb in length (14.09% of the genome), indicating that the *B. ramiflora* genome contains many unique LTR-retrotransposons. Tandem repeats were very rare, only accounting for 0.09% of the total. Unknown repeat sequences accounted for 15.91% of the total, indicating that the genome contains many new genes and unique genes. Statistical results showed that the size of the *B. ramiflora* genome was mainly determined by the LTR-retrotransposons with their repetitive sequences, which accounted for 70.91% of the total repetitive sequences and 52.1% of the total genome. In this study, a total of 3452 noncoding RNAs were found in the genome, which were mainly snRNA, rRNA, and tRNA sequences and numbered 1,981,674, and 526 respectively.

In this study, de novo, homology-based search and RNA-Seq were performed to predict genes from repeat-masked *B. ramiflora* genome sequences. Table 1 shows the final prediction results. A total of 29,172 protein-coding genes, 127.25 Mb in total length, were predicted for the *B. ramiflora* genome, with an average size of 4.4 kb per gene, generating 32,692 transcripts (52.18 Mb in total length, 1.60 kb in size on average), each gene generating an average of 1.1 transcripts. There were a total of 185,435 exons, including 178,416 coding exons (281 bp on average), each transcript containing 5.7 exons on average; there were a total of 152,743 introns (629 bp on average). The total length of coding sequences (CDS) was 40.83 Mb (1248 bp in size on average). Compared with some species of the Euphorbiaceae family, such as *R. communis* (GCA_000151685.2) (number of predicted genes: 26,049), *M. esculenta* (GCA_001659605.2) (number of predicted genes: 34,312), *H. brasiliensis* (GCA_001907995.1) (number of predicted genes: 42,686), and *J. curcas* (GCA_014843425.1) (number of predicted genes: 22,718), *B. ramiflora* has a medium number of genes, which is likely related to its high proportion of repeat sequences throughout its genome.

**Table 1:** Gene model prediction statistics in *B. ramiflora.*

Type	Value
Number of genes	29,172
Total genic length (bp)	127,254,215
Mean gene length (bp)	4362
Number of transcripts	32,692
Transcripts per gene	1.1
Total transcript length (bp)	52,174,963
Mean transcript length (bp)	1595
Number of exons	185,435
Exons per transcript	5.7
Exon length (bp)	281
Number of coding exons	178,416
Number of introns	152,743
Mean intron length (bp)	629
coding region Total CDS length (bp)	40,826,528
Average length of coding regions (mean CDS length) (bp)	1248

The comparison of gene function annotation results with those from five major databases (NR, Swiss-Prot^27^, eggNOG^28^ using DIAMOND^29^, GO using Blast2GO^30^, and KEGG^31^ using KOBAS^32^) revealed that 89.06% (25,980) of the genes were annotated, while the remaining 10.94% (3192) were not. This finding suggests that the *B. ramiflora* genome harbors many unknown genes, which implies a highly unique evolutionary process for this species. Some 25,962 (89.00%), 19,939 (68.355%), 24,699 (84.67%), 17,621 (60.40%), 8724 (9.91%) coding genes had been annotated in the NR, Swiss-Prot, eggNOG, GO, and KEGG databases, respectively. Fewer genes of *B. ramiflora* were found in the KEGG database, likely because *B. ramiflora* is a wild tree species containing many genes of unknown function.

### Phylogenetic evolution analysis

**Figure 2:**
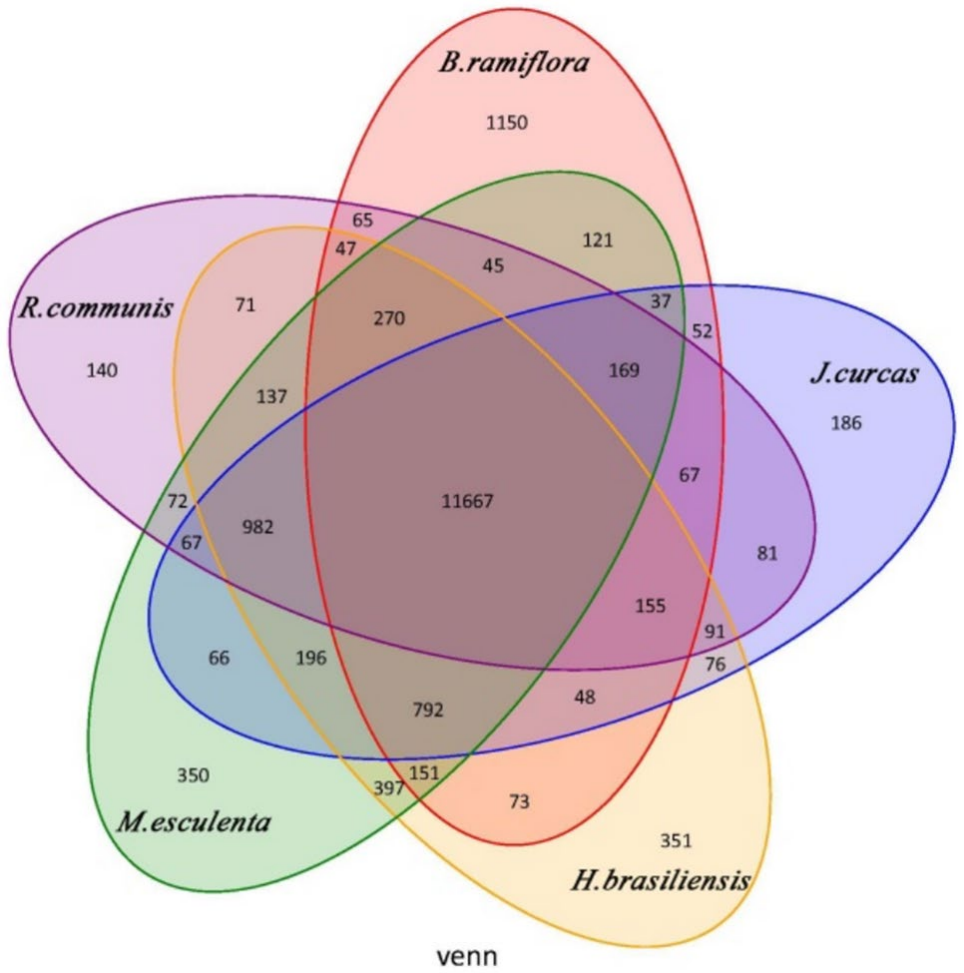
Venn diagrams of the gene families of five species of the original Euphorbiaceae.

To further understand the evolutionary position of *B. ramiflora*, we performed gene homology analysis on *B. ramiflora* and five related angiosperms (*R. communis*, *M. esculenta*, *H. brasiliensis*, *J. curcas*, and *Populus euphratica*) using *Arabidopsis thaliana* as the outgroup, the seven species containing 206,360 protein sequences, including 27,886, 18,174, 26,645, 30,653, 20,267, 28,266, and 22,938 sequences, respectively, with 3283, 304,747, 1024, 542,875, and 3081 unique protein sequences and 6267, 8440, 5836, 4836, 8214, 4407, and 6863 single-copy gene sequences (Table S1). The result of gene family cluster analysis of five species of the original Euphorbiaceae (*B. ramiflora*, *R. communis*, *M. esculenta*, *H. brasiliensis*, and *J. curcas*) is shown in Fig. 2. The common gene families contained a total of 11,667 genes, while *B. ramiflora*-specific gene families contained 1150 genes, the most among the five species, suggesting that *B. ramiflora* has acquired new gene families/genes in the evolutionary process. In *B. ramiflora*-specific gene families, 102 GO enrichment categories, mainly in catalytic activity, transferase activity, kinase activity, hydrolase activity, and nucleic acid metabolic process, were found. Thirteen enriched KEGG pathways, mainly in three aspects, i.e., genetic information (mismatch repair, homologous recombination, basal transcription factors, DNA replication, RNA degradation, nucleotide excision repair, and spliceosome), metabolic pathways (monoterpenoid biosynthesis, isoquinoline alkaloid biosynthesis, glutathione metabolism, tyrosine metabolism, alanine, aspartate, and glutamate metabolism), and plant-pathogen interaction, were found.

**Figure 3:**
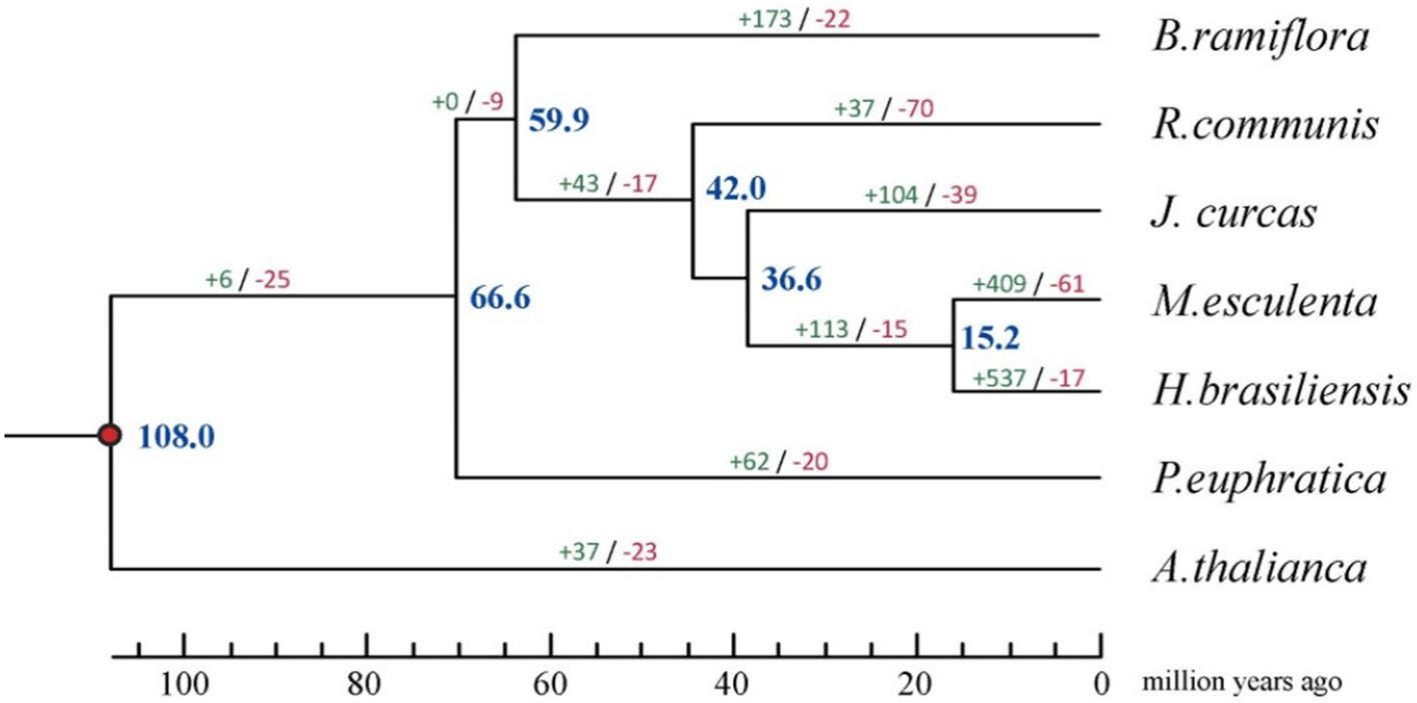
Phylogenetic tree construction of *B. ramiflora* based on single copy homologous sequences.

Clustering analysis on the gene families of *B. ramiflora*, *R. communis*, *M. esculenta*, *H. brasiliensis*, *J. curcas*, *Populus euphratica*, and *A. thaliana* was performed using the OrthoMCL method^33^, in which 1739 single-copy homologous genes were used to generate the phylogenetic tree. As shown in Fig. 3, *B. ramiflora* clustered into two sister groups with four species of the Euphorbiaceae family, which is consistent with the APG IV system, in which *B. ramiflora* belongs to the *Phyllanthus* genus. It was estimated that the divergence between *B. ramiflora* and Euphorbiaceae occurred 59.9 million years ago (Mya) (in the range of 50.4–75.2 Mya), and *B. ramiflora* and *P. euphratica* diverged from Euphorbiaceae 66.6 Mya (in the range of 53.4–75.2 Mya), while *A. thaliana* diverged from the other species 108.0 Mya (in the range of 107–109 Mya).

### Gene family expansion and contraction analysis

During the evolutionary process, 173 gene families of *B. ramiflora* expanded, mainly in metabolic pathways, including linoleic acid metabolism, monoterpenoid biosynthesis, alpha-linolenic acid metabolism, sesquiterpenoid and triterpenoid biosynthesis, glyoxylate and dicarboxylate metabolism, peroxisome, diterpenoid biosynthesis, tropane, piperidine and pyridine alkaloid biosynthesis, and anthocyanin biosynthesis; 22 gene families contracted, mainly in kinase activity, transferase activity, phosphorylation, metabolic process, and catalytic activity. Compared with four species of Euphorbiaceae, *B. ramiflora* showed nine collapsed gene families and no expanded gene families, indicating that *B. ramiflora* is closely related to the Euphorbiaceae family.

**Figure 4:**
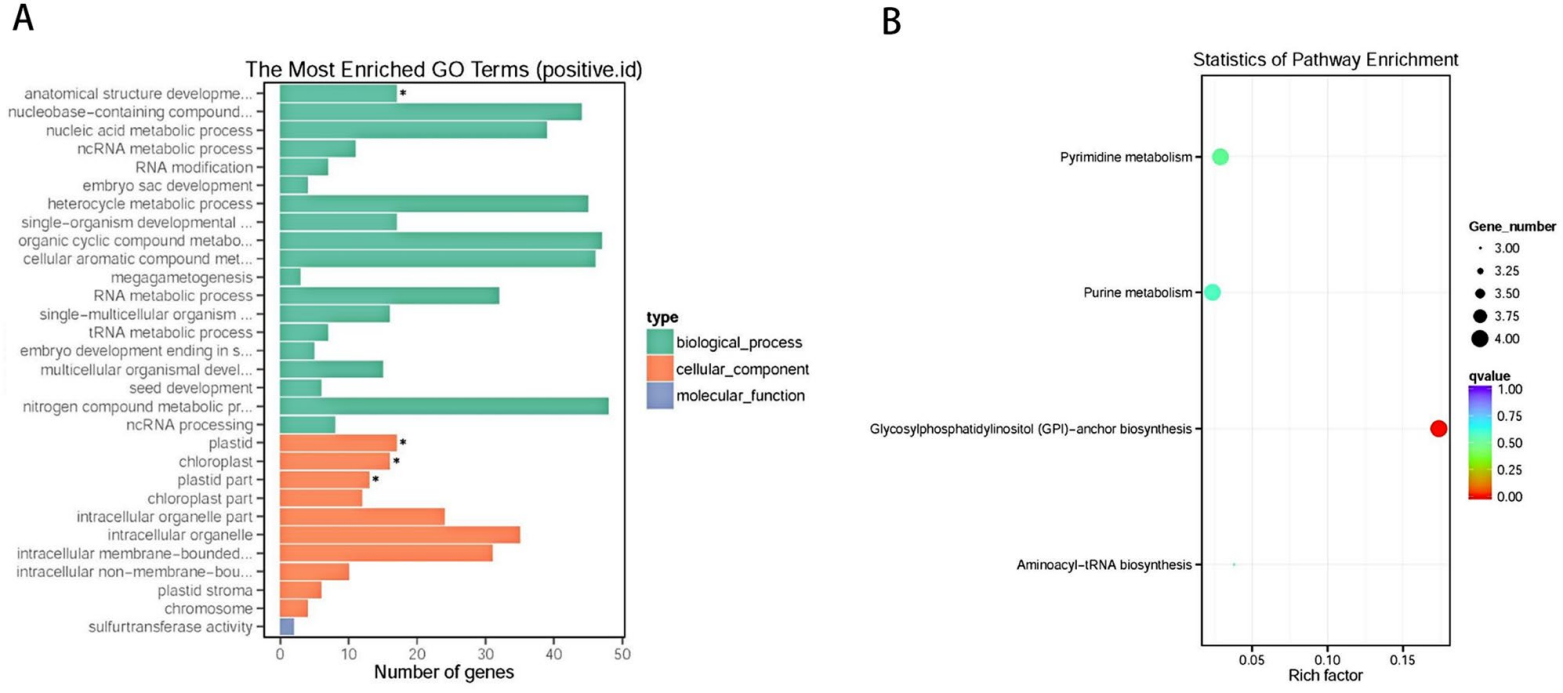
Positive selection analysis: GO enrichment (**A**) and KEGG (**B**) enrichment pathways of positive selection candidate genes.

A total of 278 candidate positive selection genes (*P* < 0.05) were screened through the likelihood ratio test and then subjected to functional enrichment analysis, which registered them to 160 GO pathway categories and four KEGG pathway categories (Fig. 4).

### Whole-genome duplication and collinearity analysis

**Figure 5:**
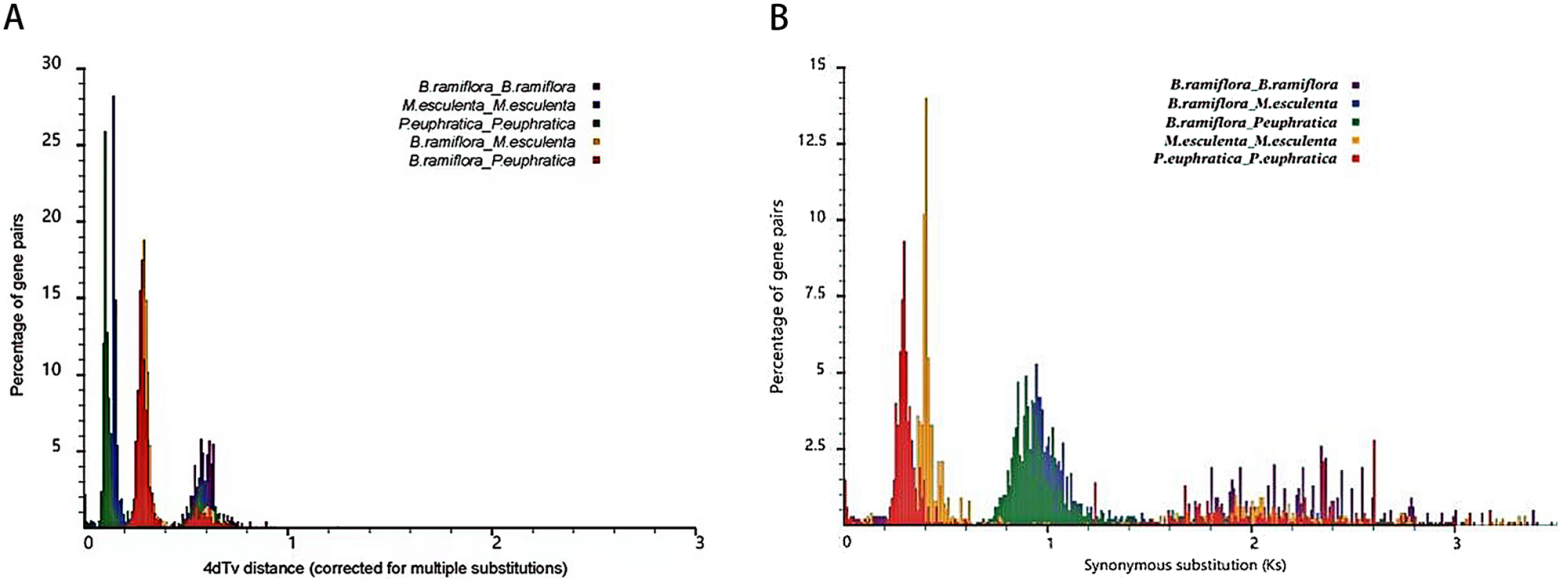
Genome-wide replication events: Pairwise *K*_s_ (**A**) and 4DTv (**B**) values of all paralogous gene pairs within *B. ramiflora.*

Whole-genome duplication (WGD) events are of great significance in plant evolution and speciation^34^. Accompanied by the doubling of genes, they provide new genes for plant evolution, accelerate the generation of new genes and the expansion of gene families, and improve the plant’s ability to adapt to environmental changes, thereby promoting plant evolution. The *K*_s_ and transversions at four-fold degenerate sites (4DTv) distribution maps of the homologous genes of *B. ramiflora*, *P. euphratica*, and *M. esculenta* (Fig. 5) showed that the *B. ramiflora* genome had one peak, corresponding to *K*_s_ and 4DTv values of 2.4 and 0.6, respectively, which were also present in both *M. esculenta* and *P. euphratica*, suggesting a genome-wide triplication event (γ) that occurred before the eudicot differentiation. No recent genomic duplication signal was detected in the *B. ramiflora* genome.

**Figure 6:**
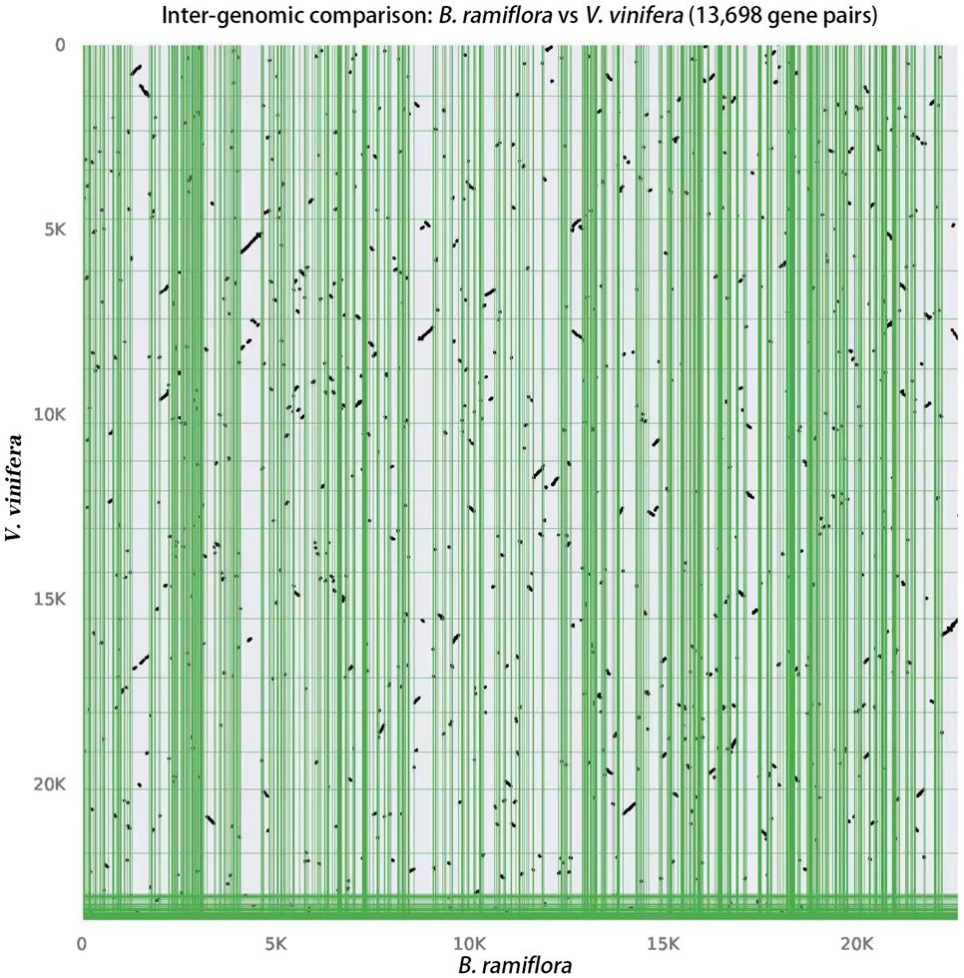
Intergenomic syntenic dot plot within *B. ramiflora* vs *V. vinifera.*

The *Vitis vinifera* genome contains chromosomal relics of its angiosperm ancestors and has been commonly adopted as a reference genome for analyzing the collinearity of angiosperm species^35^. Through collinearity alignment of the *B. ramiflora* and *V. vinifera* genomes, we found 13,698 pairs of homologous genes. As shown in the dot plot (Fig. 6), the two genomes had many collinear fragments.

However, long fragments at the same position hardly matched, suggesting that after their evolutionary divergence, no WGD events have occurred, which is consistent with the *K*_s_ and 4DTv values.

## Discussion

### Sequencing evaluation of the *B. ramiflora* genome

*Baccaurea ramiflora* is a dioecious plant with high heterozygosity and a complex genome, which makes the whole-genome sequencing and assembly of *B. ramiflora* very challenging. To sequence complex genomes with high heterozygosity and abundant repetitive sequences, it is often difficult to meet the sequencing requirements by relying solely on next-generation sequencing techniques. Third-generation sequencing uses single-molecule real-time sequencing techniques, which has the advantages of no PCR amplification and ultralong reads^36^. Therefore, in this study, we used the Pac Bio SMRT technique in combination with next-generation sequencing data to correct and assemble the sequences of the complex genome of *B. ramiflora*^37^.

Similar to whole-genome sequencing of birch (*Betula platyphylla*)^38^, we found that the long-fragment genomic DNA extracted from *B. ramiflora* had some characteristics that make third-generation sequencing advantageous, such as poor DNA integrity and low concentration, yield, and purity (e.g., viscous samples). The leaves of *B. ramiflora* contain many secondary metabolites, including polyphenols, lactones, and sterols^19,39,40^, which interfere with the extraction of long DNA fragments, and commercial DNA extraction kits do not meet the quality requirements of DNA extraction. At present, plant genome research mainly relies on methods such as DNA fragmentation removal to retain relatively integrous and longer DNA for analysis^41^. In this study, high-quality long DNA fragments we obtained and the DNA was used for the further analysis by three methods to ensure higher DNA integrity, among them Nanodrop is used to assess the purity of the DNA sample; agarose gel electrophoresis is employed to determine both the purity and integrity of DNA; Qubit is utilized for accurate quantification of DNA concentration. Additionally, during sequencing, PacBio Beads are used for purification to reduce background interference, and the some other sequences are removed from the raw data prior to the assembly process. Compared to the methods reported previously, the sequencing quality of this study still needs to be improved, and in the future, other technologies will be further employed to enhance the quality of our genome.

### Genome assembly of *B. ramiflora*

According to their heterozygosity and proportion of repetitive sequences, genomes can be divided into highly heterozygous (heterozygosity > 0.8%), mildly heterozygous (heterozygosity > 0.5%), and highly repetitive (repetitive sequences > 50%)^42^. In this study, the genome of *B. ramiflora* exhibits moderate heterozygosity and a high proportion of repeat sequences. Assembling complex genomes with high heterozygosity can pose challenges; however, the Canu + deredundancy method was successfully employed to filter out anomalous subreads. By selectively eliminating subreads with specific GC content ranges and those closely resembling mitochondrial and chloroplast sequences, we achieved a well-assembled genome with a normal distribution of GC content. Our assembly of the *B. ramiflora* genome proved highly effective compared to the assembly of the *B. platyphylla* genome^38^, which also harbors high heterozygosity. The intricacy of the *B. ramiflora* genome may account for the observed lower contig N50. Nevertheless, our assembly aligns favorably with predictions obtained from K-mer analysis, lending credence to a dependable representation of the genome devoid of overexpansion or collapse. Furthermore, validation from previous resequencing and supplementary library data provides additional support for the accuracy of our *B. ramiflora* genome assembly.

### Genome structure of *B. ramiflora*

The prediction of coding genes in the *B. ramiflora* genome was carried out using several methods including ab initio prediction, homology annotation, and RNA-seq annotation. Protein-coding genes accounting for a high level of functional annotation were predicted. Plant genomes are known to vary significantly, with factors such as transposon amplification and polyploidy events contributing to genome complexity and the increase in repetitive sequences^26,43^. In the case of *B. ramiflora*, repetitive sequences make up a high percentage of the genome, indicating a high level of repetition. The influence of LTR-retrotransposons, specifically Gypsy-type and Copia-type transposons, on repetitive sequences is also reported in previous research^44^. In addition, there are unknown repeat sequences that indicate unique conserved sequences. This may be significant for the evolution and expression of specific traits in *B. ramiflora*.

Overall, the analysis of the *B. ramiflora* genome reveals a high level of repetitive sequences, driven by transposon amplification, and suggests the presence of unique conserved sequences that may play a role in the evolution and expression of specific traits in this plant species.

### Comparative genomics of *B. ramiflora*

In this study, the phylogenetic tree of gene families was constructed for seven species, i.e., *B. ramiflora*, *R. communis*, *M. esculenta*, *H. brasiliensis*, *J. curcas*, *P. euphratica*, and *A. thaliana*. The results revealed that *B. ramiflora* grouped into two clades with species from the Euphorbiaceae family, consistent with previous phylogenetic studies^45,46^. The divergence time between *B. ramiflora* and Euphorbiaceae species indicated a close relationship between *B. ramiflora* and *P. euphratica* within the Euphorbiaceae family as sister groups. A total of 278 genes were identified to have undergone positive selection in the genome of *B. ramiflora*, suggesting the acquisition of novel genes for adaptation to its environment. Collinearity analysis indicated that these genes may have originated from remnants of an ancient whole-genome triplication event known as the γ event. Furthermore, the collinearity analysis between *B. ramiflora* and *V. vinifera* demonstrated consistent collinearity between their genomes. Following the divergence from *V. vinifera*, no further WGD events were observed in *B. ramiflora*. In terms of WGD events and collinearity, *B. ramiflora* and *V. vinifera* shared similar genomic characteristics, having experienced an ancient genome-wide triploidization event after the emergence of angiosperms, but no recent WGD events.

## Conclusion

The genome assembly of *B. ramiflora* not only provides valuable insights into its genomic characteristics, such as ornamental, edible, and medicinal value, but also has the potential to improve the reliability of other omics studies. By integrating genomics and metabolomics approaches, a more comprehensive understanding of the chemical composition and sensory attributes of this fruit can be achieved, ultimately benefiting the breeding and improvement efforts of this important tree species in the Phyllanthaceae family.

In addition, comparative genomic analysis establishes its close relationship with the Euphorbiaceae family. The identification of positive selection and the absence of recent WGD events shed light on the evolutionary history of *B. ramiflora*. This comprehensive genome resource will greatly benefit future research and tree improvement programs focused on the Phyllanthaceae family, contributing to the understanding and enhancement of this important tree species.

## Materials and methods

### Plant material

Plant material for DNA extraction was collected from young leaves of wild *B. ramiflora* from Xiexie Mountain, Lianjiang, Zhanjiang city, Guangdong Province (110°20′ 30″ E, 21°35′ 58″ N, alt 110 m) and stored in liquid nitrogen. These plant samples are also being cultivated in the greenhouse of Hanshan Normal University (116°39′58″ E, 23°39′21″ N). Crude genomic DNA was extracted from the young leaves using a cetyltrimethylammonium bromide (CTAB) method^47^, and the DNA was used for the further analysis by PacBio after strict quality control by agarose gel electrophoresis, NanoDrop One (Thermo Scientific, Shanghai, China), and Qubit (Shanghai, China), to ensure higher DNA integrity.

### Sequencing and assessment of genome size

For short-read sequencing, the 450-bp PE library was constructed using the NEBNext Ultra DNA Library Prep Kit and sequenced on the Illumina HiSeq 2500 platform. All raw Illumina sequencing reads were trimmed and filtered using Trimmomatic v0.33 to remove adapters, reads with > 3% N, and low-quality reads (those with > 50% bases with a quality score of Q < 3)^48^. For long-read sequencing, the PacBio SMRT bell library was prepared using the Template Prep Kit, then sequenced under the diffusion loading mode. Genome size was estimated through 19-bp k-mer frequency analysis^49^.

### Genome assembly and evaluation

De novo assembly was performed using Falcon v0.3.0 (length_cutoff = 5000; ength_cutoff_pr = 12,000), Canu v1.6(genomeSize = 1000 m; minReadLength = 2000), and HGAP3 (genomeSize = 1000 m; minReadLength = 2000; other default parameters) on an SGE cluster with multiple computer nodes, each consisting of 120 central processing units and 500 gigabytes of memory. The optimized configuration for the assembly was achieved using the HGAP pipeline, which then served as a reference. Finally, the Illumina data were aligned with the assembled results of the corrected third-gen data using Bwa mem v0.7.12^50^, which was followed by correction with Pilon^51^, ultimately generating the error-corrected assembly result of the third-generation and next-generation sequencing data^52^.

The assembly was evaluated through three methods, data mapping ratio evaluation, GC-Depth evaluation, and BUSCO assembly integrity evaluation. The assembly integrity assessment adopted the BUSCO method^52^, the intactness of the whole-genome sequencing assembly of *B. ramiflora* was assessed, and the completeness of the assembled genome was assessed by aligning the assembled genes of *B. ramiflora* with the conserved sequences of closely related species.

### Repeat sequence prediction

The predicted repeats in the genome were masked using RepeatMasker (v4-0-6)^53^ with the following parameters: -s -nolow -norna-gff -engine ncbi -parallel 20. The transposable elements such as miniature inverted-repeat transposable elements and LTRs were identified through the structure prediction method. Known repeat sequences of *B. ramiflora* were searched for in the RepBase library. Finally, other repeats in *B. ramiflora* genome were collected using the de novo prediction method^54^. The repeat sequences obtained through the above three methods were integrated to construct the unique repeat sequence library of *B. ramiflora*. The LTR-retrotransposon elements were identified by LTR_FINDER1.0.6^55^ to construct the de novo repeat library, and their genomic locations were determined using RepeatMasker (v4-0-6).

### Genome prediction and annotation

Protein-coding genes were annotated using a comprehensive strategy combining ab initio gene prediction, homology-based gene prediction, and Iso-Seq read-based prediction. The cDNA sequences of proteins were predicted based on homologous genes of five closely related species, *M. esculenta*, *J. curcas*, *H. brasiliensis*, *R. communis*, and *P. alba*, using GeMoMa-1.6.1. First, the genes of *B. ramiflora* were predicted de novo using GlimmerHMM, Augustus, Snap, and GeneMarkES^56,57^. The RNA-Seq reads were aligned to the scaffold sequences using HISAT2 v2.0.4^58^. The aligned reads were assembled using Cufflinks v2.2.1. Open reading frames were predicted using PASA v2.0.1^59^, of which those encoding 100–1000 AA with two or more CDSs that allowed full-length protein sequence alignment against reference sequences were selected. Gene structure was predicted with Augustus v3.0.3^56^ combined with the RNA-Seq data. Specifically, parameter training was performed on the training set, and then intron hints (i.e., the positional information of predicted introns) were obtained according to the alignment result of RNA-Seq reads and Scaffold (TopHat v2.0.10), based on which, the gene structure was predicted. Among them RNA was extracted from fresh tissues of leaves using the TruSeq RNA Sample Prep Kit (RS-122-2001). The quality of RNA was also evaluated using Nanodrop, agarose gel electrophoresis and Qubit, and the integrity of RNA was assessed using Agilent 2100. Upon passing quality control, the library was constructed and subjected to PE 150 sequencing on the Illumina Novaseq platform.

The gene structure was also predicted using SNAP and GlimmerHMM^57^. Specifically, first, parameters were trained with the training set, and then the gene structure was predicted on the scaffold with masked repeats sequences. Second, intron hints were obtained using GeneMark-ET v4.5^60^ combined with Augustus v3.0.3, based on which the gene structure of the scaffold with masked repeat sequences was predicted. Finally, the above gene prediction results were integrated using EVM^61^, and the untranslated region and alternative splicing of coding gene were predicted using PASA.

The gene annotation information was obtained by aligning with NR, Swiss-Prot, and eggNOGv4.5 using DIAMOND^29^. The GO annotation was performed using Blast2GO, so the predicted genes were matched to their respective GO numbers. The KEGG annotation was performed using KOBAS 2.0^32^ to obtain functional annotation of gene products. By individually aligning the genes to each of the five major databases, the corresponding gene function annotation results were obtained, and then the gene function annotation results were integrated to draw Venn diagrams to generate more accurate gene function annotation information.

### Contract and expansion of *B. ramiflora* gene families and phylogenetic analysis

Homologous gene clustering analysis was conducted on *B. ramiflora* and five closely related species with well-annotated genomes, i.e., *R. communis*, *M. esculenta*, *H. brasiliensis*, and *J. curcas*, and *P. euphratica*, taking *A. thaliana* as the outgroup species and applying the OrthoMCL method^33^. Specifically, the protein sequences of the above seven species were selected, of which the transcripts with the longest alternatively spliced coding regions were retained, while those coding for shorter than 50 amino acids were excluded, and then the protein-coding sequences were determined with all-vs.-all BLAST alignment, with the e value set to 1e-5, to calculate the similarity between the sequences. The clustering analysis was performed using the Markov clustering algorithm. The clustering result of protein families was shown in a Venn diagram. Unique gene families of *B. ramiflora* were obtained through protein family cluster analysis. The expansion and contract of gene families of the above-described seven species in the evolutionary process were analyzed in Cafe software.

Based on the clustering results of protein families, phylogenetic analysis of *B. ramiflora* was performed. Specifically, single-copy gene families of the seven species were selected as reference markers, and supergenes were constructed through 4DTv. Multiple-sequence alignment on the seven species was performed using Mafft v7.294^62^, and the poorly matched regions were filtered using Gblocks v.0.91b^63^ to select the most suitable base substitution model to obtain sequence files with better alignment results. The confidence of each branch was tested using Rax ML v8.0.19^64^, the GTRGAMMA model in Maximum Likelihood (ML)^65^ and 1000 bootstrap runs before the phylogenetic analysis. The divergence time was estimated based on phylogenetic analysis, by extracting the fourfold degenerate sites through the CDS alignment of single-copy gene family sequences and then generating complete sequences according to the order of species and genes. The divergence time was estimated using the MCMCtree program of PAML v4.6 software^66^, with the following parameters: burn in = 10,000; sample number = 100,000; sample frequency = 2; clock = 2. In reference to the known differentiation times of other species in the database of the Time Tree^67^, the divergence time of *B. ramiflora* was estimated.

### Whole-genome duplication event and collinearity analysis

The protein sequences of homologous genes of *B. ramiflora*, *P. euphratica*, and *M. esculenta* were pairwise aligned, and the ratio of nonsynonymous substitution rate (*K*_a_) over the synonymous substitution rate (*K*_s_) (ω = Ka/Ks) was calculated using the KaKs_Calculator v.2.0 software^68^, and the 4D sites and 4DTv values of homologous genes were analyzed to obtain the genes with more than 20 sites. The divergence events between *B. ramiflora* and other species and their respective WGD events were predicted based on the 4DTv values. The collinear regions within *B. ramiflora* genome and the collinearity between *B. ramiflora* and *V. vinifera* were detected by MCScan v0.8^69^ with the rule that each collinear region contain at least 20 homologous genes in the same order, based on which the collinearity map was generated. The multiple CDSs of single-copy gene families of the seven species were used for alignment, and the branch site–specific model in the codeml tool of PAML was used to detect whether each gene family had been positively selected in the subbranch of *B. ramiflora*, based on the ω value of the coding sequence (if ω > 1, then the gene family has undergone positive selection), which was then tested based on the likelihood ratio of the two hypotheses.

### Supplementary Information

Supplementary Information.

## Data Availability

All data has been uploaded to NCBI GenBank (PRJNA845134) and will be made available online via repository upon acceptance. Additional information on the methods are available online at, and can be cited directly by doi:.

## References

[1] Li PT (1994). Euphorbiaceae. Flora China.

[2] Goyal AK, Mishra T, Sen A (2013). Antioxidant profling of Latkan (*B. ramifora* Lour.) wine. Indian J. Biotechnol..

[3] Bhowmick N (2011). Some lesser known minor fruit crops of northern parts of West Bengal. Acta. Hortic..

[4] Wang HJ, Xing YQ, Lin S, Luo ZW, Gu YF (2013). Research and application of *Baccaurea ramiflora* fruit resources. Mod. Agric. Sci. Tech..

[5] Chen J (2023). Identification of key taste components in *Baccaurea ramiflora* Lour. fruit using non-targeted metabolomics. Food Sci. Hum. Well..

[6] Hu JX, Xiao CF, Zheng LL (2003). *Baccaurea ramiflora*: A wild fruit tree. South Chin. Fruits.

[7] Luo PS (2014). Investigation on the germplasm resources and selection of elite individual plants of *Baccaurea ramiflora* in Guangxi. South Chin. Fruits.

[8] Li WX (2015). Determination of soluble saccharide contents of *Baccaurea ramilfora* Lour. using anthrone colorimetric method. Chin. Hortic. Abstr..

[9] Pandey Y, Upadhyay S, Bhatt SS, Sharma L, Chanbisana C (2018). Nutritional compositions of *Baccaurea sapida* and *Eleaocarpus sikkimnesis* of Sikkim Himalaya. Int. J. Curr. Microbiol. Appl. Sci..

[10] Inta A, Trisonthi P, Trisonthi C (2013). Analysis of traditional knowledge in medicinal plants used by Yuan in Thailand. J. Ethnopharmacol..

[11] Lin YF, Yi Z, Zhao CH (2003). Color Atlas of Dai Medicine in China.

[12] Rahim ZB, Rahman MM, Saha D, Hosen SMZ, Paul S, Kader S (2012). Ethnomedicinal plants used against jaundice in Bangladesh and its economical prospects. Bull Pharm. Res..

[13] Kalita D, Saikia J, Mukherjee AK, Doley R (2014). An ethnomedicinal survey of traditionally used medicinal plants for the treatment of snakebite in Moriga on district of Assam India. Int. J. Med. Aroma. Plants..

[14] Saha S, Gouda TS, Srinivas SV (2017). Preliminary phytochemical analysis and oral acute toxicity study of the leaves of *Baccaurea ramifora* and *Microcos paniculata*. Saudi. J. Med. Pharm. Sci..

[15] Usha T, Middha SK, Bhattacharya M, Lokesh P, Goyal A (2014). Rosmarinic acid a new polyphenol from *Baccaurea ramiflora* Lour. leaf: A probable compound for its anti-infammatory activity. Antioxidants.

[16] Mann S, Sharma A, Biswas S, Gupta RK (2015). Identifcation and molecular docking analysis of active ingredients with medicinal properties from edible *Baccaurea sapida*. Bioinformation..

[17] Pan ZH, Ning DS, Huang SS, Wu YF, Ding T, Luo L (2015). A new picrotoxane sesquiterpene from the berries of *Baccaurea ramifora* with antifungal activity against *Colletotrichum gloeosporioides*. Nat. Prod. Res..

[18] Saha MR, Dey P, Chaudhuri TK, Goyal AK, Sen A (2016). Assessment of haemolytic cytotoxic and free radical scavenging activities of an underutilized fruit *Baccaurea ramifora* Lour. (Roxb.) Muell. Indian J. Exp. Biol..

[19] Usha T, Pradhan S, Goyal AK, Dhivya S, Middha SK (2017). Molecular simulation-based combinatorial modeling and antioxidant activities of Zingiberaceae family rhizomes. Pharmacogn. Mag..

[20] Nesa ML (2018). Screening of *Baccaurea ramifora* (Lour.) extracts for cytotoxic analgesic anti-infammatory neuropharmacological and antidiarrheal activities. BMC. Complem. Altern. M..

[21] Koren S, Walenz BP, Berlin K, Miller JR, Bergman NH, Phillippy AM (2017). Canu: Scalable and accurate long-read assembly via adaptive κ-mer weighting and repeat separation. Genome Res..

[22] Ruan J, Li H (2020). Fast and accurate long-read assembly with wtdbg2. Nat Methods..

[23] Kolmogorov M, Yuan J, Lin Y, Pevzner PA (2019). Assembly of long, error-prone reads using repeat graphs. Nat. Biotechnol..

[24] Daccord N (2017). High-quality *de novo* assembly of the apple genome and methylome dynamics of early fruit development. Nat. Genet..

[25] Ouyang S, Buell R (2004). The TIGR plant repeat databases: A collective resource for the identification of repetitive sequences in plants. Nucleic Acids Res..

[26] Xu L, Zhang Y, Su Y, Liu L, Yang J, Li ZC (2010). Structure and evolution of full-length LTR retrotransposons in rice genome. Plant Syst. Evol..

[27] Bairoch A, Apweiler R (2000). The SWISS-PROT protein sequence database and its supplement TrEMBL in 2000. Nucleic Acids Res..

[28] Huerta-Cepas J (2017). Fast genome-wide functional annotation through orthology assignment by eggNOG-mapper. Mol. Biol. Evol..

[29] Buchfink B, Xie C, Huson DH (2015). Fast and sensitive protein alignment using DIAMOND. Nat. Methods.

[30] Conesa A, Götz S (2008). Blast2GO: A comprehensive suite for functional analysis in plant genomics. Int J Plant Genom..

[31] Kanehisa M, Sato Y, Kawashima M (2022). KEGG mapping tools for uncovering hidden features in biological data. Protein Sci.

[32] Xie C (2011). KOBAS 2.0: A web server for annotation and identification of enriched pathways and diseases. Nucleic Acids. Res..

[33] Li L, Stoeckert CJ, Roos DS (2003). OrthoMCL: Identification of ortholog groups for eukaryotic genomes. Genome Res..

[34] Haas M (2021). Whole-genome assembly and annotation of northern wild rice, *Zizania palustris* L., supports a whole-genome duplication in the Zizania genus. Plant J..

[35] Shi X (2023). The complete reference genome for grapevine (*Vitis vinifera* L.) genetics and breeding. Hortic. Res..

[36] Liu YH, Wang L, Yu L (2015). Principle and application of single-molecule real-time sequencing technology. Hereditas..

[37] Yang Y, Du XJ, Liang B, Guo JD, Cheng XZ, Wang S (2015). Development of third-generation sequencing technologies and related bioinformatics. Food Res. Develop..

[38] Wang S (2019). Whole Genome Sequencing and Analysis of *Baccaurea platyphylla* (*B. platyphylla*).

[39] Xu J, Lin Q, Liang ZY, Deng SM, Zhong CX (2007). Study on chemical constituents of essential oils from *Baccaurea ramiflora* Lour. fruit, leaf and root. Food Sci..

[40] Ning DS, Wu YF, Lv SH (2014). Chemical constituents of the stem and leaves of *Baccaurea ramiflora*. Guihaia..

[41] Cui C, Herlihy JH, Bombarely A, McDowell JM, Haak DC (2019). Draft assembly of *Phytophthora capsici* from long-read sequencing uncovers complexity. Mol. Plant Microbe Interact..

[42] Wu YTN, Du HY, Li FD (2014). Advances in Whole Genome Sequencing of *Eucommia ulmoides*.

[43] Moghe GD, Shiu SH (2014). The causes and molecular consequences of polyploidy in flowering plants. Ann. N. Y. Acad. Sci..

[44] Storz G (2002). An expanding universe of noncoding RNAs. Science.

[45] Li J, Gao X, Sang S, Liu C (2019). Genome-wide identification, phylogeny, and expression analysis of the SBP-box gene family in Euphorbiaceae. BMC Genom..

[46] Prananda AT (2023). *Phyllanthus emblica*: A comprehensive review of its phytochemical composition and pharmacological properties. Front Pharmacol..

[47] Schenk JJ, Becklund LE, Carey SJ, Fabre PP (2023). What is the “Modified” CTAB protocol? Characterizing modifications to the CTAB DNA extraction protocol. Appl. Plant Sci..

[48] Vurture GW (2017). GenomeScope: Fast reference-free genome profiling from short reads. Bioinformatics.

[49] Koren S (2018). De novo assembly of haplotype-resolved genomes with trio binning. Nat. Biotechnol..

[50] Li, H. Aligning sequence reads, clone sequences and assembly contigs with BWA-MEM. *Genomics* **1303**, 1–3 (2013).

[51] Walker BJ (2014). Pilon: An integrated tool for comprehensive microbial variant detection and genome assembly improvement. PLoS ONE.

[52] Seppey M, Manni M, Zdobnov EM (2019). BUSCO: Assessing genome assembly and annotation completeness. Methods Mol. Biol..

[53] Tarailo-Graovac M, Chen N (2009). Using RepeatMasker to identify repetitive elements in genomic sequences. Curr. Protoc. Bioinform..

[54] Price AL, Jones NC, De Pevzner PA (2005). *novo* identification of repeat families in large genomes. Bioinformatics.

[55] Xu Z, Wang H (2007). LTR_FINDER: An efficient tool for the prediction of full-length LTR retrotransposons. Nucleic Acids Res..

[56] Mario S, Rasmus S, Stephan W, Burkhard M (2004). AUGUSTUS: A web server for gene finding in eukaryotes. Nucleic Acids Res..

[57] Majoros WH, Pertea M, Salzberg SL (2004). TigrScan and GlimmerHMM: Two open source ab initio eukaryotic gene-finders. Bioinformatics.

[58] Kim D, Langmead B, Salzberg SL (2015). HISAT: A fast spliced aligner with low memory requirements. Nat. Methods.

[59] Roberts A, Pimentel H, Trapnell C, Pachter L (2011). Identification of novel transcripts in annotated genomes using RNA-Seq. Bioinformatics.

[60] Ter-Hovhannisyan V, Lomsadze A, Chernoff YO, Borodovsky M (2008). Gene prediction in novel fungal genomes using an ab initio algorithm with unsupervised training. Genome Res..

[61] Haas BJ (2008). Automated eukaryotic gene structure annotation using evidence modeler and the program to assemble spliced alignments. Genome Biol..

[62] Katoh K, Standley DM (2013). MAFFT multiple sequence alignment software version 7: Improvements in performance and usability. Mol. Biol. Evol..

[63] Castresana J (2000). Selection of conserved blocks from multiple alignments for their use in phylogenetic analysis. Mol. Biol. Evol..

[64] Stamatakis A (2006). RAx ML-VI-HPC: Maximum likelihood-based phylogenetic analyses with thousands of taxa and mixed models. Bioinformatics..

[65] Ronquist F, Huelsenbeck JP (2003). Mr Bayes 3: Bayesian phylogenetic inference under mixed models. Bioinformatics..

[66] Yang Z (2007). PAML 4: Phylogenetic analysis by maximum likelihood. Mol. Biol. Evol..

[67] Hedges SB, Dudley J, Kumar S (2006). Time tree: A public knowledge-base of divergence times among organisms. Bioinformatics.

[68] Zhang Z, Li J, Zhao XQ, Wang J, Wong KS, Yu J (2006). Ka Ks_Calculator: Calculating Ka and Ks through model selection and model averaging. Genome Proteom. Bioinform..

[69] Tang H, Bowers JE, Wang X, Ming R, Alam M, Paterson AH (2008). Synteny and collinearity in plant genomes. Science.

