# Supplementary material for: Genome assembly provides insights into the genome evolution of Baccaurea ramiflora Lour
Source: Sci Rep. 2024 Feb 28;14:4867. doi: 10.1038/s41598-024-55498-4 (PMC10901894; doi:10.1038/s41598-024-55498-4)
Supplement: Supplementary file 1 — Supplementary Information. [file 41598_2024_55498_MOESM1_ESM.pdf]

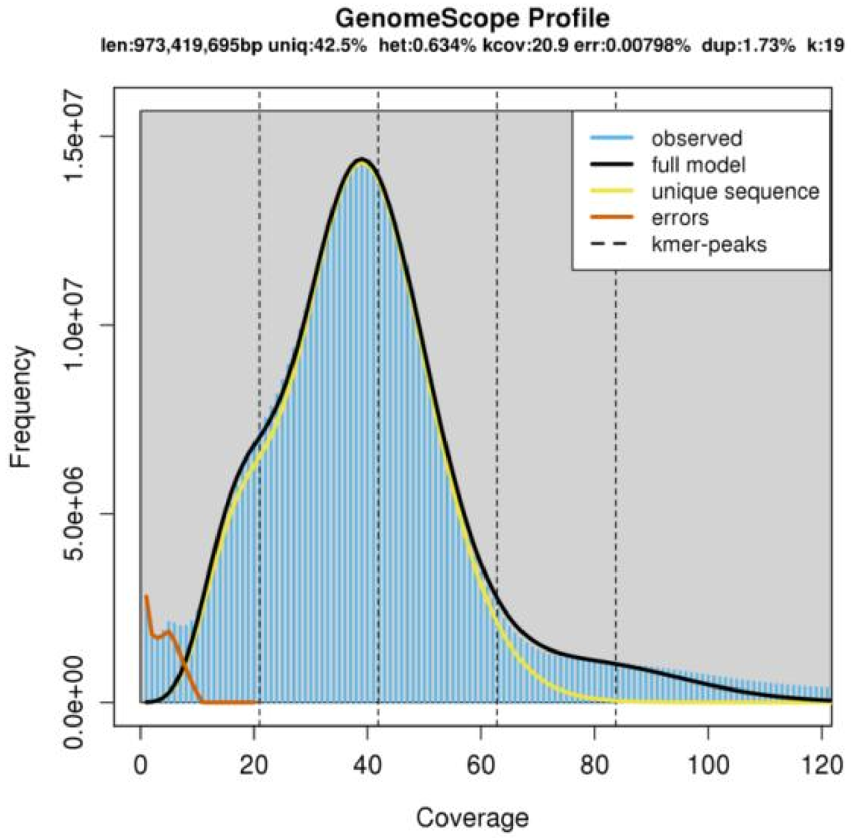

Figure S1 19-mer distribution curve of the genome of *Baccaurea ramiflora*

Table S1 Genomic assembly statistics in *Baccaurea ramiflora*

Assembly methods	Genome size (Mb)	Contig N50 (Mb)	DNA Mapping rate and properly	Busco assessment	GCdepth analysis
CANU + deredundancy	945	1.200	96.05%;69.59	C:88.7%[S:61.9%,D:26.8%], F:1.4%,M:9.9%,n:2121	no abnormal peaks
WTDBG	1,300	0.710	96.71%;80.05%	C:91.8%[S:87.7%,D:4.1%], F:1.5%,M:6.7%,n:2121	abnormal peaks
WTDBG + deredundancy	919	0.950	95.99%;71.73%	C:90.6%[S:86.8%,D:3.8%], F:1.7%,M:7.7%,n:2121	abnormal peaks
Flye + deredundancy	1,010	0.397	98.28%;85.82%	C:97.2%[S:82.2%,D:15.0%], F:0.9%,M:1.9%,n:2121	abnormal peaks
Canu（filtered the original subreads）	985	0.300	96.86%;80.27%	C:95.4%[S:81.9%,D:10.2%], F:1.3%,M:2.1%,n:2121	no abnormal peaks
Canu（filter out the subreads highly homologous）	973	0.503	98.86%;81.42%	C:96.7%[S:85.1%,D:11.6%], F:1.1%,M:2.2%,n:2121	no abnormal peaks

Table S2 Comparison between RNA-seq data and assembly results

Sample Name	Reads Number	Mapping Rate	Concordantly0	Concordantly1	Concordantly >1
Root	30,563,210	88.38%	5,733,970;18.76%	14,953,466;48.93%	9875774;10.99%
Stem	24,505,473	89.73%	4389943;17.91%	12,306,017;50.22%	7809513;31.87%
Leaf	29,533,881	90.43%	5031113;17.04%	15,069,818;51.03%	9432950;31.94%

Note: Concordantly0, the ratio of Reads to different chromosomes or not meeting the requirements of inserted fragments; Concordantly1, Reads were paired to the same chromosome once and meet the requirement of the length of the inserted fragment; Concorplanar >1, Reads are pair-to-pair aligned to the same chromosome multiple times and meet the requirement for the length of the inserted fragment.

Table S3 Statistical of repeated sequences in the genome of *Baccaurea ramiflora*

Family	Repeat type	Classification	Number	Masked (bp)	Masked (%)
Class I:	LTR-Retrotransposon	Copia	105,655	89,672,164	9.19%
		Gypsy	233,054	281,215,828	28.82%
		Others	251,655	137,528,549	14.09%
	Non-LTR Retrotransposon	LINE	26,666	16,477,327	1.69%
Class II: DNA	Subclass I	CMC-EnSpm	4,131	4,298,089	0.44%
		Maverick	103	22,394	0.00%
		MULE-MuDR	11,864	10,371,377	1.06%
		PIF-Harbinger	1,215	725,409	0.07%
		hAT-Ac	2,729	1,282,578	0.13%
		TcMar-Stowaway	138	40,387	0.00%
		hAT-Tip100	1,239	413,528	0.04%
		Others	75,918	17,750,231	1.82%
	Subclass II	Helitron	986	661,926	0.07%
	Tandem Repeat	Simple repeat	3,824	846,506	0.09%
	Unknown	-	648,953	155,225,579	15.91%
	Total	-	1,368,735	716,928,090	73.47%

Table S4 Gene homology analysis among the seven plant species

Plant species	Protein sequences	unique protein sequences	single-copy gene sequences
Baccaurea ramiflora	27,886	3,283	6,267
Ricinus communis	18,174	304	8,440
Manihot esculenta	26,645	747	5,836
Hevea brasiliensis	30,653	1,024	4,836
Jatropha curcas	20,267	542	8,214
Populus euphratica	28,266	875	4,407
Arabidopsis thaliana	22,938	3,081	6,863
Manihot esculenta	26,645	747	5,836
